# Leucine minimizes denervation-induced skeletal muscle atrophy of rats through akt/mtor signaling pathways

**DOI:** 10.3389/fphys.2015.00073

**Published:** 2015-03-18

**Authors:** Carolina B. Ribeiro, Daiane C. Christofoletti, Vitor A. Pezolato, Rita de Cássia Marqueti Durigan, Jonato Prestes, Ramires A. Tibana, Elaine C. L. Pereira, Ivo V. de Sousa Neto, João L. Q. Durigan, Carlos A. da Silva

**Affiliations:** ^1^Programa de Pós-graduação em Ciências do Movimento Humano, Methodist University of Piracicaba, UNIMEPPiracicaba, Brazil; ^2^Graduate Program of Science and Technology of Health, University of BrasíliaBrasilia, Brazil; ^3^Graduate Program of Physical Education, Catholic University of BrasíliaBrasilia, Brazil; ^4^Graduate Program of Physical Education, University of BrasíliaBrasilia, Brazil

**Keywords:** denervation, leucine, muscle atrophy, synthesis pathways, degradation pathways, rehabilitation

## Abstract

The aim of the present study was to evaluate the effect of leucine treatment (0.30 mM) on muscle weight and signaling of myoproteins related to synthesis and degradation pathways of soleus muscle following seven days of complete sciatic nerve lesion. Wistar rats (*n* = 24) of 3–4 months of age (192 ± 23 g) were used. The animals were randomly distributed into four experimental groups (*n* = 6/group): control, treated with leucine (L), denervated (D) and denervated treated with leucine (DL). Dependent measures were proteins levels of AKT, AMPK, mTOR, and ACC performed by Western blot. Leucine induced a reduction in the phosphorylation of AMPK (*p* < 0.05) by 16% in the L and by 68% in the DL groups as compared with control group. Denervation increased AMPK by 24% in the D group as compared with the control group (*p* < 0.05). AKT was also modulated by denervation and leucine treatment, highlighted by the elevation of AKT phosphorylation in the D (65%), L (98%) and DL (146%) groups as compared with the control group (*p* < 0.05). AKT phosphorylation was 49% higher in the D group as compared with the DL group. Furthermore, denervation decreased mTOR phosphorylation by 29% in the D group as compared with the control group. However, leucine treatment induced an increase of 49% in the phosphorylation of mTOR in the L group as compared with the control group, and an increase of 154% in the DL as compared with the D group (*p* < 0.05). ACC phosphorylation was 20% greater in the D group than the control group. Furthermore, ACC in the soleus was 22% lower in the in the L group and 50% lower in the DL group than the respective control group (*p* < 0.05). In conclusion, leucine treatment minimized the deleterious effects of denervation on rat soleus muscle by increasing anabolic (AKT and mTOR) and decreasing catabolic (AMPK) pathways. These results may be interesting for muscle recovery following acute denervation, which may contribute to musculoskeletal rehabilitation after denervation.

## Introduction

Peripheral nerve injuries are commonly present during clinical practice of orthopedics and traumatology, in which alterations of the functional pattern of peripheral and central nervous system are accompanied by muscle tissue disuse. In case of total interruption of motor innervation, there is an immediate loss of tissue voluntary and reflex action, intramuscular proliferation of connective tissue, and decrease or loss of muscle force generating capacity (Reardon et al., 2001; Dow et al., 2006; Durigan et al., 2014).

During denervation there is an increase of muscle proteins related to proteolysis, such as atrogin-1/MAFbx and MuRF1 (Muscle RING Finger 1), as well as the reduction in the expression of proteins related to protein synthesis, such as AKT/mTOR (serine/threonine-specific protein kinase and Mammalian Target of Rapamycin, respectively), which in turns modulate the 4EBP1 p70S6K1 (Bodine et al., 2001; Lima et al., 2007; Salvini et al., 2012; Bonaldo and Sandri, 2013). AMP-activated protein kinase (AMPK), a sensor of cellular energy status, was recently shown to increase myofibrillar protein degradation and autophagy through the expression of MAFbx and MuRF1 (Sanchez et al., 2012).

Consequently, skeletal muscle undergoes in a harmful nutrition status with modification of acetyl-CoA carboxylase (ACC) activity (Han et al., 2007), as well as a peripheral insulin resistance due to the decrease of phosphatidylinositol 3-kinase (PI3K) levels, which is linked to the insulin receptor and the reduction of glucose transporters (GLUTs). This process results in the decrease of energetic reserves and muscle proteolysis (Coderre et al., 1992; Bodine et al., 2001; Salvini et al., 2012; Bonaldo and Sandri, 2013). Some substances have been used as a therapeutic strategy to minimize denervation induced atrophy, such as branched-chain amino acids (BCAAs), especially leucine (Kelleher et al., 2013). It was demonstrated that the use of leucine-enriched high protein diet do not protect soleus single fiber characteristics (fiber size and contractile function) after 60-day bed rest in humans. The divergent response in size and functional MHC I soleus properties with the concurrent exercise program was a unique finding further highlighting the challenges of protecting the unloaded soleus, maintaing MHC power but not size and strength (Trappe et al., 2008). Mounier et al. (2009) also demonstrate that the nutrition of proteins and BCAAs countermeasure was more specifically efficient against slow fiber atrophy assessed by western blotting and force of single skinned fibers after a 60-day bed rest.

It has been shown that BCAA prevents dexamethasone-induced muscle atrophy in rats (Yamamoto et al., 2010). Other studies have shown that BCAA inhibited atrogin-1 and MuRF1 expression in the C2C12 mouse muscle cell line (Xu et al., 2001; Shimomura et al., 2006; Herningtyas et al., 2008; Yamamoto et al., 2010), and minimized the muscle atrophy in rats that were immobilized for 7 weeks (Xu et al., 2001; Shimomura et al., 2006; Herningtyas et al., 2008; Yamamoto et al., 2010; Kelleher et al., 2013). In addition, leucine treatment can attenuate the loss of lean mass, improve tissue repair and muscle protein turnover in elderly individuals, and induce beneficial effects for the treatment of liver and kidney diseases (Norton and Layman, 2006; Maki et al., 2012; Kelleher et al., 2013).

Taking together, these findings suggest that BCAA may protect against muscle atrophy by inhibiting protein breakdown as well as protein synthesis. However, the effects of leucine on the atrophy process and correlated signaling pathways during the acute phase of denervation are not known. Thus, we aimed to evaluate the effects of leucine treatment on muscle weight and signaling for synthesis and degradation of myoproteins in the soleus muscle of rats following total injury of the sciatic nerve. We hypothesized that leucine treatment would minimize the deleterious effects of denervation in the soleus muscle.

## Methods

Wistar rats (*n* = 24) of 3–4 months of age (192 ± 23 g) were used. The animals housed in collective polypropylene cages in a room with controlled temperature ranging (22–27°C) on a 12 h light/dark cycle and had free access to water and a balanced diet with standard rodent chow (Purina, Descalvado-São Paulo, Brazil). The animal experiments described here were approved by the Institutional Committee for Ethics in Animal Experimentation (protocol n 011/2006) and were done in accordance with the guidelines of the Brazilian College for Animal Experimentation (COBEA).

The animals were randomly distributed into four experimental groups (*n* = 6/group): control, treated with leucine (L), denervated (D) and denervated treated with leucine (DL). Both the period of denervation and treatment lasted 7 days. Leucine was delivered with doses of 0.30 mM/100g (Sigma St. Louis, MO, USA) introduced into the digestive system via an orogastric flexible tube. A dose-response curve based in a previous study of our laboratory revealed that 0.30 mM was effective in maintaining muscle glycogen content after denervation. After anesthesia with sodium tiopental (40 mg/kg body weight, ip), muscles were denervated by surgical removal of 1 cm of sciatic nerve from left posterior hindlimb (Coderre et al., 1992).

Following 7 days of treatment, the animals were anesthetized in a CO_2_ chamber and euthanized. The soleus muscle was rapidly removed, washed in saline solution, weighted and mixed to an extraction buffer, as follow: (100 mM Trisma base, pH 7.5, 10 mM EDTA, 100 mM sodium pyrophosphate, 100 mM NaF, 10 mM Na_3_VO_4_, 2 mM PMSF diluted in ethylic alcohol, 1% Triton X-100 and 0.1 mg/ml aprotinine), at 4°C in a Polytron PTA 20S homogenizer (model PT 10/35; Brinkmann Instruments, Westbury, NY) at maximal velocity for 30 s. The cell fragments were centrifuged (15.500 × g, 20 minutes, 4°C) for the removal of the insoluble material, and the supenatant was used for the assay. Part of the material was used for the determination of total protein content by the Biuret photo colorimetric method (Bradford), while the remaining material was used for Western Blot analysis. The reagents for the determination of total protein content (Biuret), SDS/PAGE and Western Blot were from Bio-Rad (Richmond, CA, USA). Nitrocellulose membranes were from Amersham Corp. (Aylesbury, UK). The chemiluminescence kit was obtained from Endogen (Rockford, IL). All other materials were obtained from Sigma (St. Louis, MO, USA).

After the measurement of total protein content, a buffer containing 100 mM of dithiothreitol was added to the supernatant, and then heated for 5–10 min. Equal quantities of proteins (75 μg) were submitted to eletroforesis in poliacrilamide SDS-PAGE gels at the BIO-RAD miniature slab gel apparatus (Mini-Protean, Bio-Rad Laboratories, Richmond, CA). The electrotransfer of gel proteins to the membrane lasted 120 min at 120 V in a miniaturized transference device (BIO-RAD). The binding of the antibodies to non-specific proteins was reduced by the pre-incubation of the membrane with blocking buffer under room temperature (5% BSA dissolved in basal solution) to 120 min. The nitrocelulose membrane was incubated, overnight, with specific antibodies: anti-pSer473AKT (#4058), anti-pAMPK (#2537), anti-pmTOR (#2971), and anti-pACC (#3661) obtained from Cell Signaling Technology (Beverly, MA), diluted in antibody solution (3% BSA); and then washed for 15 min with basal solution (150 mM NaCl, 10 mM Trisma base and 0.02% of Tween 20). Detection of the antigen-antibody complex attached to the nitrocelulose membrane was determined via chemiluminescence by using a commercial kit from Amersham and following recommendations from the manufacturer. β-Actin was used as the internal control. Following the revelation of the auto-radiographs, the identified bands were quantified by optical densitometry.

## Statistical analysis

All variables showed normal distribution (Shapiro–Wilk test) and homoscedasticity (Levene test). Thus, Two-Way ANOVA (denervation x treatment) followed by the Tukey multiple paired analysis was used for comparisons among treatments. Differences were considered significant when *P* < 0.05. All data were analyzed using the Statistica 7.0 software package (StatSoft Inc., Tulsa, OK, USA).

## Results

There were group × time interactions showing that denervation and leucine treatment interact with muscle weight (*p* < 0.05). Denervation decreased soleus muscle weight by 35.1% as compared with the control group (*p* < 0.05). Leucine treatment minimized muscle weight loss by 28% in the DL group as compared with the D group (*p* < 0.05). Moreover, soleus muscle weight was 17.5% greater in the L group than the control group (*p* < 0.05; Table 1).

**Table 1:** **Soleus muscle mass/body mass ratio (MM/BM) in the control group (C), treated with leucine (L), denervated (D), and denervated treated with leucine (DL)**.

**Groups**	**MM/BM**
C	0.55 ± 0.01
L	0.65 ± 0.01^*^
D	0.36 ± 0.04^*^
DL	0.46 ± 0.008^*^^#^^~^

*Values are mean ± standard error of the mean (SEM), n = 6*.

* p< 0.05 compared to C,

# p < 0.05 compared to D;

~ *p < 0.05 compared to L*.

**Figure 1:**
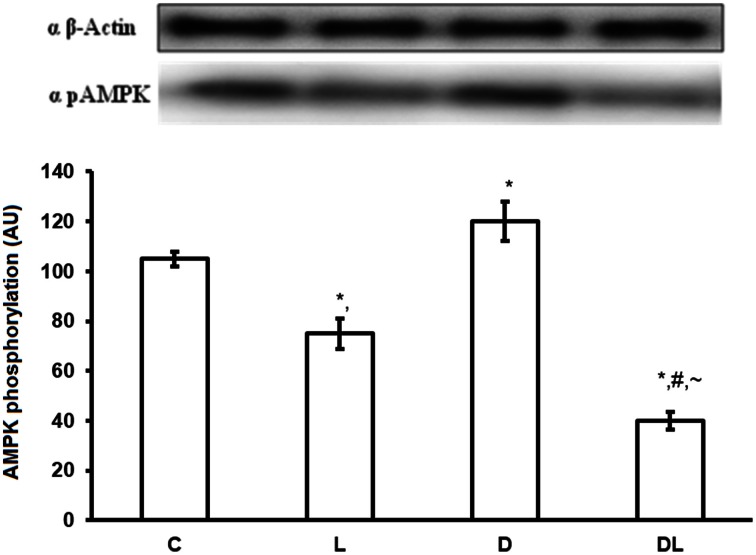
**AMPK protein phosphorylation in arbitrary unit (AU) in the control group (C), treated with leucine (L), denervated (D), and denervated treated with leucine (DL)**. Values are mean ± standard error of the mean (SEM), *n* = 6. ^*^p < 0.05 compared to C, ^#^*p* < 0.05 compared to D; ~*p* < 0.05 compared to L.

**Figure 2:**
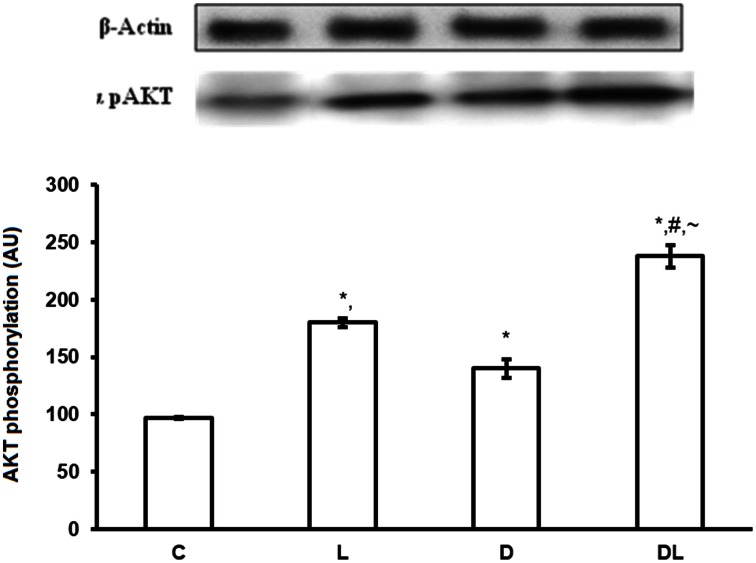
**AKT protein phosphorylation in arbitrary unit (AU) in the control group (C), treated with leucine (L), denervated (D), and denervated treated with leucine (DL)**. Values are mean ± standard error of the mean (SEM), *n* = 6. ^*^p < 0.05 compared to C, ^#^*p* < 0.05 compared to D; ~*p* < 0.05 compared to L.

There were group × time interactions showing that denervation and leucine treatment interact with to the AMPK, AKT, mTOR, and ACC proteins level. Leucine induced a reduction in the phosphorylation of AMPK (*p* < 0.05) by 16% in the L and by 68% in the DL groups as compared with control group. Denervation increased AMPK by 24% in the D group as compared with the control group (*p* < 0.05; Figure 1). AKT was also modulated by denervation and leucine treatment, highlighted by the elevation of AKT phosphorylation in the D (65%), L (98%), and DL (146%) groups as compared with the control group (*p* < 0.05). AKT phosphorylation was 49% higher in the D group as compared with the DL group (Figure 2; *p* < 0.05).

**Figure 3:**
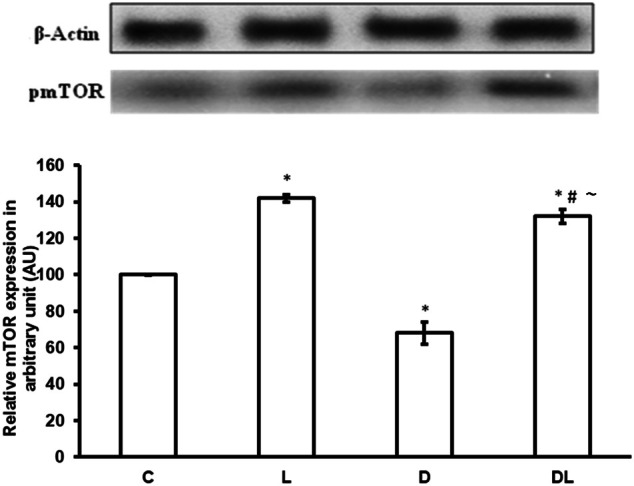
**mTOR protein phosphorylation in arbitrary unit (AU) in the control group (C), treated with leucine (L), denervated (D), and denervated treated with leucine (DL)**. Values are mean ± standard error of the mean (SEM), *n* = 6. ^*^p < 0.05 compared to C, ^#^*p* < 0.05 compared to D; ~*p* < 0.05 compared to L.

**Figure 4:**
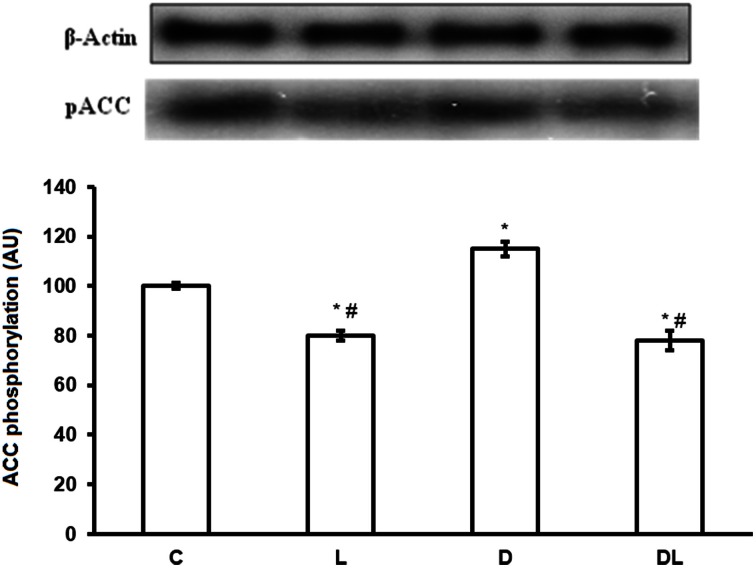
**ACC protein phosphorylation in arbitrary unit (AU) in the control group (C), treated with leucine (L), denervated (D), and denervated treated with leucine (DL)**. Values are mean ± standard error of the mean (SEM), *n* = 6. ^*^p < 0.05 compared to C, ^#^*p* < 0.05 compared to D.

Furthermore, denervation decreased mTOR phosphorylation by 29% in the D group as compared with the control group. However, leucine treatment induced an increase of 49% in the phosphorylation of mTOR in the L group as compared with the control group, and an increase of 154% in the DL as compared with the D group (Figure 3; *p* < 0.05). ACC phosphorylation was 20% greater in the D group than the control group. Furthermore, ACC in the soleus was 22% lower in the in the L group and 50% lower in the DL group than the respective control group (Figure 4; *p* < 0.05).

## Discussion

To the best of our knowledge, this is the first study to investigate the effects of leucine treatment following 7 days of denervation on the molecular pathways of muscle protein synthesis and degradation of rats. The initial hypothesis was confirmed, since leucine minimized the early deleterious effects of denervation on the soleus muscle by increasing important molecular pathways involved with muscle protein synthesis. Although this study was performed in animals, it has clinical relevance and indicates the importance of leucine treatment in the acute phase of muscle atrophy with the attempt to reduce the deleterious effects related to denervated muscles.

Leucine is known to regulate protein turnover of muscle cells, inhibiting protein degradation and promoting protein synthesis (Norton and Layman, 2006; Tom and Nair, 2006; Maki et al., 2012; Kelleher et al., 2013). Among the possible mechanisms involved, leucine can activate protein synthesis in the muscle, heart, liver and kidney by mTOR-dependent pathways (mTOR, S6K1, and 4E-BP1) and mTOR-independent pathways (Norton and Layman, 2006). Moreover, it has been shown that the ability of leucine to phosphorylate p70^s6k^ (serine/threonine kinase that acts downstream PI3-K pathway) is blocked by rapamycin, an inhibitor of mTOR and phosphatidylinositol 3-kinase (PI3-K) (Tom and Nair, 2006).

Escobar et al. (2006), found that leucine increases the phosphorylation of S6K1 and rpS6 in glycolytic muscles, such as the type II myosin containing latissimus dorsi. Furthermore, the elevation of plasma leucine induced an increase in the phosphorylation of 4E-BP1 at the Thr^70^, with a concomitant decrease of 4E-BP1 and eIF4E complex; and an increase of the eF4G complex and IF4E in skeletal muscle (Escobar et al., 2006). Similarly, the present study revealed that leucine treatment increased the phosphorylation of AKT, mTOR and reduced ACC in the soleus muscle following 7 days of denervation, indicating the anti-catabolic effect of leucine during denervation-induced atrophy.

Leucine also know to affect the autophagy-lysosome system. Mordier et al. (2000) showed that leucine restriction induces the formation of autophagy and activation of lysosomal-dependent proteolysis in C2C12 myotube. Kadowaki and Kanazawa (2003) suggest that leucine has direct regulatory potencial in liver autophagic proteolysis. In addition, a reduction in muscle mass was suppressed by leucine-supplementation (1,5% leucine) in rats fed protein-free diet for 7 days. The results showed that the expression of a very specific marker of autophagen, microtubule-associated protein light chain 3 active form (LC3-II) was significantly decreased, wherein atrogin-1 and MuRF1 were not suppressed. It is suggested that inhibition of autophagy-lysossome is likely the system involved in the suppression of myofibrillar protein degradation by leucine (Sugawara et al., 2009).

Interestingly, our results revealed that denervation decreased mTOR and increased AMPK and ACC, which are activated during energy catabolism, such as decreased ATP levels. It has been proposed that p70^s6k^ controls AMPK signaling and affects intracellular energetic metabolism, represented by the AMPK/ATP ratio. Although leucine may modulate the AMPK/ATP ratio and mTOR/p70^s6k^ pathways, the increase of AMPK phosphorylation may occur without a relevant modification of muscle energy status (Deshmukh et al., 2009; Iwanaka et al., 2010). This fact may explain the reduction of mTOR signaling and consequently a reduction of muscle atrophy observed in denervated and treated animals.

Futhermore, Filiputti et al. (2010) investigated the islet cells of rats treated with different diets, and found that leucine treatment increased mTOR activation by 2.5 times in a group with diet restriction (composed of 6% of protein) and in the normal diet group. Leucine treatment also minimized the decrease of S6K-1 ribosomal protein induced by protein restriction. These results indicate that the activation of PI-3K and mTOR induced by leucine may be involved in insulin secretion during protein restriction.

In the present study, denervation increased AMP-activated protein kinase expression, indicating that the autophagy protein breakdown system may be involved in muscle atrophy induced by denervation. Along this line, Sugawara et al. (2009) showed that L-leucine reversed a reduction in gastrocnemius muscle weight and reversed increased LC3II expression (an autophagy marker) induced by a protein-free diet for 7 days in rats. The difference between their study and ours is likely due to the differences in the experimental methods. They selected a protein-free diet for an induction of muscle atrophy, which markedly increases autophagy, while we demonstrated, for the first time, the role of AMP-activated protein kinase expression after denervation-induced atrophy. Our results suggest that leucine treatment may protect muscle mass via the autophagy pathway, and increasing AKT/mTOR signaling pathways.

It is important to consider some limitations of the present study. Movement restriction inherit to the denervation surgery process could exert some influence on the results of the denervation groups, possibly potentiating the effects of disuse atrophy, especially during the first few days of denervation. Future studies should consider a follow up of animals to access pain and movement restriction during the first days after the denervation process and correlate these finds with muscle disuse adaptation. Moreover, we have a limitation of AKT normalization in the western blotting assay. This may explain the surprising increase of AKT in the phosphorylated states of denervated muscles. Future studies should also include non-phosphorylated forms to normalize the phospho isoforms of AKT and also include different AKT isoforms. Moreover, antiphosphatases inhibitors should be used to prevent degradation of the phosphoisoforms of AKT and mTOR. Muscle cross sectional area analysis should also be included. The results revealed atrophy in the entire soleus muscle, and selective atrophy on different types of muscle fibers could be missed.

## Conclusion

In conclusion, leucine treatment minimized the deleterious effects of denervation on rat soleus muscle by increasing anabolic (AKT and mTOR) and decreasing catabolic (AMPK) pathways. These results may be interesting for muscle recovery following acute denervation. Future studies investigating additional molecular pathways, muscle function and morphology should be conducted.

### Conflict of interest statement

The authors declare that the research was conducted in the absence of any commercial or financial relationships that could be construed as a potential conflict of interest.
